# Epstein–Barr Virus-Positive Diffuse Large B cell Lymphoma in the Experience of a Tertiary Medical Center in Poland

**DOI:** 10.1007/s00005-015-0341-2

**Published:** 2015-06-18

**Authors:** Mateusz Ziarkiewicz, Dominika Wołosz, Tomasz Dzieciątkowski, Ewa Wilczek, Jadwiga Dwilewicz-Trojaczek, Wiesław Wiktor Jędrzejczak, Beata Gierej, Bogna Ziarkiewicz-Wróblewska

**Affiliations:** Department of Hematology, Oncology and Internal Medicine, Medical University of Warsaw, Warsaw, Poland; Department of Pathology, Center for Biostructure Research, Medical University of Warsaw, Warsaw, Poland; Department of Microbiology, Central Clinical Hospital, Warsaw, Poland; Chair and Department of Medical Microbiology, Medical University of Warsaw, Warsaw, Poland

**Keywords:** Diffuse large B cell lymphoma, DLBCL, Epstein–Barr virus, EBV, EBV-positive DLBCL of the elderly, EBER, EBV serology

## Abstract

The role of Epstein–Barr virus (EBV) in the biology and clinical characteristics of diffuse large B cell lymphoma (DLBCL) is still poorly defined. A new provisional entity EBV-positive DLBCL of the elderly has been described in Asian population. Its incidence and prognosis remains unknown in middle European patients. Clinical data and tissue samples were collected from 74 Caucasian patients with DLBCL, aged between 23 and 86 years, treated at a single institution. Lymphoma morphology was reassessed, laboratory procedures included in situ hybridization specific for EBV-encoded small RNAs (EBER), immunohistochemical staining for latent membrane protein and serological testing for EBV-specific antibodies. EBER staining revealed 12.2 % of EBV-positive cases, whereas 9.5 % were diagnosed as EBV-positive DLBCL of the elderly. Serologic EBV markers did not correlate with the presence of EBV in tissue samples (*P* > 0.10). Elderly EBV-positive cases had lower BCL-6 (*P* = 0.038) and higher CD30 (*P* = 0.049) expression and were characterized by higher progression risk (median time-to-progression 12.5 months vs not reached; *P* = 0.029) and a trend towards worse overall survival (median overall survival 24.5 months vs not reached; *P* = 0.059). EBV-positive DLBCL of the elderly occurs relatively frequently in Polish population and may be associated with inferior prognosis in comparison with DLBCL, not otherwise specified.

## Introduction

Epstein–Barr virus (EBV) is a member of γ-herpesvirinae subfamily, one of the most important groups of oncogenic viruses (Cohen et al. 2008; Rezk and Weiss 2007; Schuster and Muschen 2003). It has been isolated for the first time by Michael Epstein and Yvonne Barr in 1964 from the Burkitt lymphoma cell line (Epstein et al. 1965). EBV causes both acute and chronic infections, with seropositivity in IgG class reaching 90 % of the adult population. It infects both epithelial cells and lymphocytes, with special inclination to CD21^high^ B memory cells, which constitute the viral reservoir in latent infections. Three latency programs have been proposed in different B cell differentiation stages according to the expression of latent viral antigens, namely latency type III (growth program) in naive B cells, latency type II, (default program) in germinal center B cells (GCB), and latency type I in memory B cells. All EBV-infected cells contain large amounts of small non-coding fragments of viral RNA (EBV-encoded small RNAs: EBER) that are useful for clinical diagnosis (Rezk and Weiss 2007).

EBV has been proposed as important cofactor in the development of multiple malignancies. Viral latent membrane proteins (LMPs) have documented oncogenic potential. LMP1 belonging to the tumor necrosis factor receptor family is an autonomic activator of signaling pathway, which leads subsequently to activation of transcription factor NF-κB and antiapoptotic protein BCL-2 (Vockerodt et al. 2011). EBV has central role in lymphomagenesis in immunocompromised individuals, being the causative factor of post-transplant lymphoproliferative disorders and important cofactor in HIV-related lymphomas. In immunocompetent individuals, EBV is involved in the pathogenesis of Burkitt lymphoma, Hodgkin lymphoma, rare lymphomas of large B cells, as well as some T and NK cell lymphomas.

The role of EBV in diffuse large B cell lymphoma (DLBCL) has become a discussed issue in recent years. DLBCL constitutes a very heterogeneous disease with multiple poorly defined subtypes. Clinical studies show inferior prognosis of patients with EBV-positive DLBCL. World Health Organization (WHO) 2008 classification of lymphoid malignancies includes a new provisional entity EBV-positive DLBCL of the elderly (Nakamura et al. 2008). This DLBCL subtype is defined as a disease of people older than 50 years without any known immunodeficiency, characterized by advanced clinical stage and more frequent cutaneous and pulmonary localization, and signs of latent EBV infection (Oyama et al. 2007). However, EBV-positive DLBCL of the elderly is mainly described in Asian populations, with lower or unknown incidence in Caucasians (Gibson and Hsi 2009; Hoeller et al. 2010).

The aim of the present study was to examine the incidence of EBV infection in a historical cohort of consecutive DLBCL cases treated at a single center (Medical University of Warsaw, Warsaw, Poland), with special emphasis on EBV-positive DLBCL of the elderly subtype, and to test the prognostic impact of EBV-related parameters.

## Materials and Methods

### Patients

The study was a retrospective analysis of 74 patients with the diagnosis of DLBCL treated at the Department of Hematology, Oncology and Internal Medicine of the Medical University of Warsaw (Poland) between 1994 and 2011. Inclusion criteria were the histological diagnosis of DLBCL according to WHO 2008 classification, as well as availability of clinical data and paraffin-embedded biopsy specimens. All patients with secondary or transformed DLBCL, as well as with other large B cell lymphomas (especially primary mediastinal lymphoma or plasmablastic lymphoma) were excluded from the study. The study was performed in accordance with the Helsinki Declaration of 1975, as revised in 2000 and 2008 and received approval from the Institutional Review Board at the Medical University of Warsaw.

### Histological and Serological Procedures

All pathologic specimens were reviewed by two pathologists with expertise (BZW and BG) and reclassified in accordance with the WHO criteria for pathologic diagnosis. Immunohistochemical analysis was performed on paraffin sections using antibodies specific for CD3, CD20, CD10, CD5, CD30, LMP1, Ki67, MUM-1, BCL-2, BCL-6, manufactured by Dako (Denmark).

Percentages of positive cells were counted as a mean value from ten high-power fields at magnification 200×. According to CD10, BCL-6 and MUM-1 expression, DLBCL was further categorized into GCB and non-GCB subtype, using the algorithm proposed by Hans et al. (2004).

EBV RNA was detected by an in situ hybridization (ISH) technique (Rembrandt, PanPath, Netherlands). The paraffin-embedded 4-μm sections were dewaxed with xylene followed by treatment with pepsin digestion. Hybridization procedure was performed with digoxigenin-labeled RNA oligonucleotides complementary to EBER. As a control both negative and positive control probes were used. For the probe detection anti-digoxigenin–horseradish peroxidase conjugate was applied. Staining was visualized using 3,3′-diaminobenzidine (DAB) as a chromogen. Subsequently, slides were counterstained with hematoxylin. A positive reaction was defined as more than 5 % nuclear positivity of cells with malignant morphology.

Levels of antibodies specific for EBV, both in IgM and IgG classes, were measured in a panel of patients’ serum specimens, using a commercial quantitative ELISA test against EBV antigens: VCA, EA and EBNA-1 (IBL, Germany), according to the manufacturer’s instruction. Threshold serum activity for positivity was set at 8.0 IU/ml for all serological assays, encompassing both borderline and strongly positive cases (cutoff value provided by the manufacturer, IBL).

Viral DNA was detected with real-time PCR assay, using commercial quantitative EBV Quant Kit^®^ in samples extracted from the 200 μl of serum, using a High Pure Viral Nucleic Acid Kit^®^, in accordance with the manufacturer’s guidelines. Tests were run on the LightCycler 2.0 instrument and each amplification reaction embraced, except tested samples and calibrators, a negative control of DNA extraction and amplification process. All reagents and instruments were supplied by Roche Diagnostics, Germany.

### Treatment

Patients received a median of 8 cycles of anthracycline-based polichemotherapy (CHOP or its variants; *n* = 61, 82.4 %), or non-anthracycline-based regimens (*n* = 13, 17.6 %). Chemotherapy was combined with rituximab in 53 (71.9 %) patients. Median number of treatment lines was 1 (range 1–5). Eight patients (10.8 %) underwent consolidation therapy with autologous stem cell transplantation, 7 patients (9.4 %) were treated with adjuvant radiotherapy and 12 patients (16.2 %) received intrathecal injections of cytostatic agents as prophylaxis or treatment of central nervous system involvement.

### Statistical Analysis

Overall survival (OS), progression-free survival (PFS) and time-to-progression (TTP) were calculated using the Kaplan–Meier estimator. OS was defined as the period from the date of diagnosis to the date of death from any cause or the last follow-up. PFS was defined as the period from the date of diagnosis to the date of the first-documented disease relapse or progression, death, or the last follow-up. TTP was defined as the period from the date of diagnosis to the date of the first-documented disease relapse or progression, or the last follow-up. Survival estimates in specific patients groups were compared for statistical differences using log-rank test. Other comparisons were performed using the non-parametric *U* Mann–Whitney’s method. *P* values less than 0.05 were considered statistically significant. All *P* values correspond to two-sided significance tests.

## Results

### Patient Characteristics

Seventy four patients with the histological diagnosis of DLBCL were included in the study. All patients were Caucasians, aged between 23 and 86 years. Half of the patients were male (*n* = 37). None of the patients was immunocompromised; all patients were HIV-seronegative. Most important baseline characteristics of the study population according to age and EBER expression are presented in Table 1.

**Table 1:** Baseline patient characteristics according to EBER status and age

Parameter	Overall (*n* = 74)	All ages			Age ≥50		
		EBER^−^ (*n* = 65)	EBER^+^ (*n* = 9)	*P*	EBER^−^ (*n* = 44)	EBER^+^ (*n* = 7)	*P*
Median age, years	63.5	60.3	57.9	NS	71.5	64.5	NS
Sex							
Males, *n* (%)	37 (50.0)	32 (49.2)	5 (55.5)	NS	18 (40.9)	3 (42.9)	NS
Females, *n* (%)	37 (50.0)	33 (50.8)	4 (44.5)		26 (59.1)	4 (57.1)	
Ann Arbor stage ≤2, *n* (%)	29 (40.8)	27 (41.5)	2 (25.0)	NS	18 (41.9)	2 (28.6)	NS
B symptoms, *n* (%)	43 (62.3)	36 (55.4)	7 (77.8)	NS	23 (57.5)	6(85.7)	NS
Mediastinal bulk ≥50 mm, *n* (%)	6 (9.0)	6 (9.2)	0 (0)	NS	3 (7.5)	0 (0)	NS
Bone marrow involvement, *n* (%)	10 (14.7)	8 (12.9)	2 (22.2)	NS	5 (11.9)	1 (14.3)	NS
ECOG ≥2, *n* (%)	22 (32.4)	19 (29.2)	3 (33.3)	NS	17 (41.5)	3 (42.9)	NS
LDH ≥ ULN, *n* (%)	46 (71.9)	39 (60.0)	7 (77.8)	NS	27 (69.2)	7 (100)	NS
IPI 0–2, *n* (%)	41 (58.6)	38 (58.5)	3 (33.3)	NS	16 (41.0)	2 (28.6)	NS
Rituximab-based chemotherapy, *n* (%)	49 (68.1)	46 (70.8)	3 (33.3)	NS	29 (65.9)	2 (28.6)	NS
GCB, *n* (%)	21 (30.4)	19 (29.2)	2 (22.2)	NS	11 (26.2)	2 (28.6)	NS

*EBER* EBV-encoded small RNA, *ECOG* performance status scale, *LDH* lactate dehydrogenase, *ULN* upper limit of normal, *IPI* international prognostic index, *GCB* germinal center B cell subtype, *LMP1* latent membrane protein 1, *NS* not significant

All studied cases had available paraffin-embedded tissue material for EBER ISH analysis. In 54 patients (73.0 %) pre-treatment titers of EBV-specific antibodies were available. Quantification of EBV DNA in sera specimens was performed only in four cases (5.4 %) with negative results in all tested samples. Therefore, EBV DNA data were not included in further analysis.

### EBER and LMP1 Results

EBER ISH procedure gave positive nuclear reaction in 9 out of 74 cases (12.2 %). The threshold for positivity was set at 5 % of malignant cells. In our series nuclear-positive reaction was found in 5–40 % of malignant cells. IHC staining for LMP1 gave positive membranous reaction in 6 out of 70 evaluable cases (8.6 %). Both EBER and LMP1 were tested for significant associations with clinical and pathological factors related with patient status, staging and biologic tumor characteristics. In the present study, none of the clinical parameters differ significantly according to EBER or LMP expression. Similarly, there were no clinical differences between the subgroup classified as EBV-positive DLBCL of the elderly and age-matched EBER-negative DLBCL cases (*P* ≥ 0.10 for both comparisons; see Table 1). Amongst pathologic tumor characteristics, none was associated with EBER or LMP, but BCL-6 and CD30 expression was different in EBV-positive DLBCL of the elderly (see below). Patients grouped according to EBER status or the diagnosis of EBV-positive DLBCL of the elderly did not differ significantly in terms of treatment intensity or rituximab use (*P* = 0.237 and *P* = 0.132, respectively), even though the percentage of rituximab-containing regimens was lower in EBV-positive cases (see Table 1).

Interestingly, no significant association between EBER and LMP1 positivity was noted in the study group (3/9 EBER-positive patients had positive LMP1 results and 3/6 LMP1-positive patients had positive EBER result, *P* = 0.139).

### EBV Serology

**Fig. 1:**
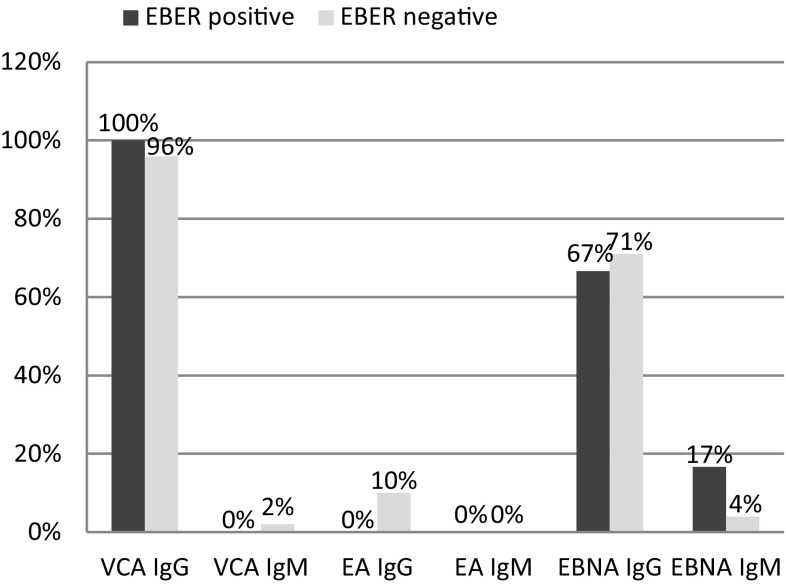
Frequencies of serum EBV-specific antibodies in relation to EBER status (*P* > 0.05 for all categories). *EBER* EBN-encoded small RNA, *VCA* viral capsid antigen, *EA* early antigen, *EBNA* EBV nuclear antigen

In a subset of 54 patients (73.0 %) baseline serum titers of antibodies specific for viral capsid antigen (VCA), early antigen (EA) and EBV nuclear antigen (EBNA-1) in both IgG and IgM classes were available for analysis. Figure 1 depicts the relative frequencies of serologic and histological EBV markers. VCA IgG and EBNA IgG were present in the great majority of tested samples, whereas EA IgG were only rarely found. IgM positivity was found in 0, 1.9, and 7.9 % of IgG-positive cases for EA, VCA and EBNA, respectively. None of the serum EBV-specific antibodies was significantly associated with the presence of EBV in DLBCL clone, measured either by LMP1 or EBER (*P* = 0.516). Similarly, an independent analysis of IgM antibodies, irrespective of antigen specificity, revealed no associations with LMP1 or EBER (*P* = 0.628 and *P* = 0.644, respectively). Moreover, the diagnosis of EBV-positive DLBCL of the elderly was not associated with any of the analyzed serological profiles (data not shown).

Presence of IgM antibodies against both VCA and EBNA was associated with younger age (mean age 28.9 vs 62.9 years; *P* = 0.001). Moreover, the titer of EBNA IgG antibodies declined with age (mean age 55.4 vs 72.0 in EBNA IgG-positive and EBNA IgG-negative group, respectively; *P* < 0.001). EBNA-positive cases were characterized by less frequent localized extranodal involvement in comparison with EBNA-negative cases (22 vs 66.0 %; *P* = 0.012).

Analysis of cases with serologic features of acute EBV infection or reactivation of chronic infection [EA IgG/IgM (+); VCA IgG (+) VCA IgM (+) EBNA IgG (±), 9.3 % (5/54)], as well as chronic EBV infection [VCA IgG (+) VCA IgM (−) EBNA IgG (+), 68.5 % (37/54)] did not add any new information to the data described above. There was no association between serologically defined acute or chronic EBV infection and EBER or LMP1 status. Acute EBV infection was not associated with age, whereas chronic EBV infection appeared to be linked with younger age, probably due to the decline of EBNA IgG in older DLBCL patients (see above).

### EBV-Positive DLBCL of the Elderly

**Fig. 2:**
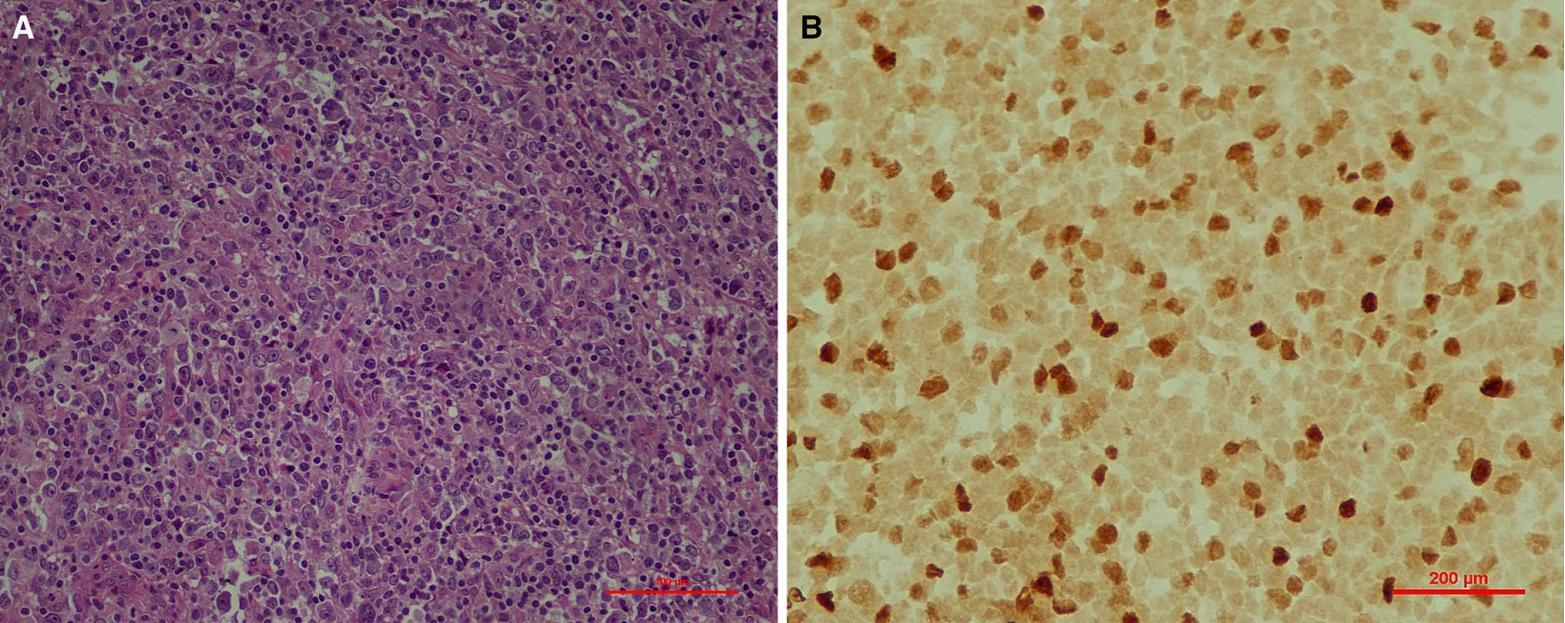
EBV-positive diffuse large B cell lymphoma of the elderly. *Red bar* 200 μm. **a** HE staining, **b** EBER in situ hybridization

Seven out of nine EBER-positive patients (9.5 % of all 74 patients in the study) had no identified immunodeficiency state and were over 50 years of age and thus were classified as EBV-positive DLBCL of the elderly. Clinicopathologic characteristics of this subgroup is summarized in Table 2. Figure 2 depicts the morphology and EBER staining in a representative case from the study. Mean age of the patients was 64.5 years. Mean clinical stage according to Ann Arbor was 3 and all but one case had B symptoms. Extranodal involvement occurred in three cases (42.9 %), namely invasion of the kidney and the gastrointestinal tract, lungs, and the bone marrow. All EBV-positive DLBCL of the elderly patients had elevated serum baseline lactate dehydrogenase (LDH) activity and four of seven cases (57.1 %) had high or intermediate risk according to international prognostic index (IPI). The morphology of malignant cells was centroblastic in six cases (85.7 %) and anaplastic with large Reed–Sternberg-like cells in one case. The histological picture was monomorphic in four (57.1 %) and polymorphic with abundant inflammatory infiltrate in three cases (42.9 %). The infiltrates in polymorphic cases included plasma cells, histiocytes and lymphocytes. In two cases signs of fibrosis were observed, whereas none showed signs of necrosis. Phenotype of malignant cells was non-GCB in five cases (71.4 %) and GCB in two cases (28.6 %). All cases were CD20 positive; mean ki-67 proliferative index was 70 %. The expression of BCL-6 on tumor cells was significantly lower (mean percentage of BCL-6-positive cells 0 vs 34.0 % in EBV-positive DLBCL of the elderly vs other cases; *P* = 0.038). The mean expression of CD30 was 29 % in EBV-positive DLBCL of the elderly cases in comparison to 12 % in the remaining population (*P* = 0.049). Immunohistochemical expression of CD10, MUM-1, CD5, BCL-2 and ki-67 was comparable between both patient groups. Four patients were EBER positive and LMP1 negative and only two patients had both EBER and LMP1 expression.

**Table 2:** Characteristics of cases classified as EBV-positive diffuse large B cell lymphoma of the elderly

Patient	A	B	C	D	E	F	G
Age	50.7	55.1	55.3	65.2	70.4	77.0	77.8
Stage	4B	3B	4B	4B	2A	2B	3B
Extranodal involvement	Lungs	–	BM	–	–	GI, kidney	–
LDH (U/L)	U	794	1544	505	390	1360	1639
IPI	U	3	2	3	2	3	4
Morphologic variant	Poly	Poly	Mono	Poly	Mono	Mono	Mono
Plasma cells	−	+	−	+	−	−	−
Cell of origin	Non-GCB	Non-GCB	Non-GCB	Non-GCB	GCB	Non-GCB	GCB
Ki-67 (%)	70	70	50	90	50	80	80
BCL-6	−	−	−	−	−	−	−
CD30	+	+	+	+	−	+	−
EBER (%)	5	40	5	5	10	5	5
LMP1	U	+	+	−	−	−	−

*GI* gastrointestinal tract, *BM* bone marrow, *Mono* monomorphic, *Poly* polymorphic, *GCB* germinal center B cell subtype, *U* unknown, *LDH* lactate dehydrogenase, *IPI* international prognostic index, *LMP1* latent membrane protein 1

### Survival Analysis

**Fig. 3:**
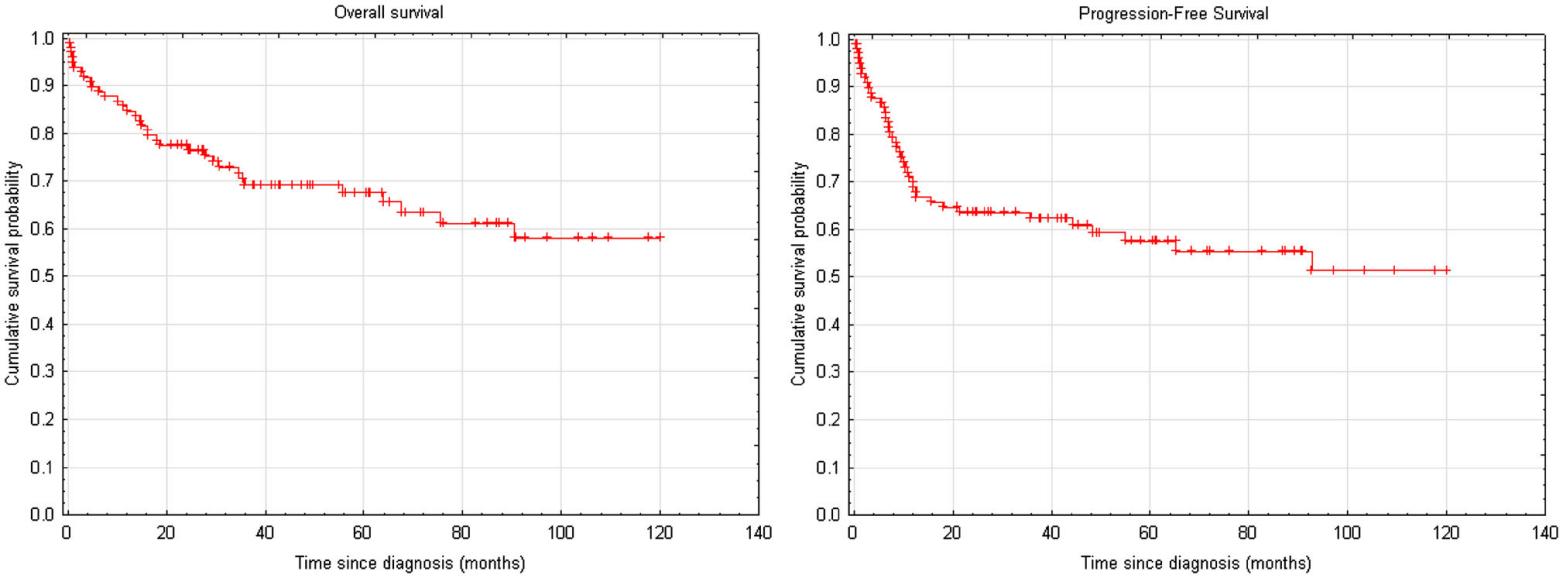
Overall survival and progression-free survival of the study group

After a median follow-up duration of 44.4 months (range 0.4–291.3), median OS and PFS in the study population have not been reached. According to Kaplan–Meier estimator, 1-, 5- and 10-year OS probability were 84.7, 67.6 and 58.1 %, respectively (see Fig. 3a). One, five and ten-year PFS probability reached 69.8, 57.9, 51.3 %, respectively (see Fig. 3b). TTP analysis revealed cumulative progression probability of 22.2, 32.4 and 39.2 % at 1, 5 and 10 years, respectively. Median TTP for the whole study population has not been reached. In the subgroup of 33 (33.7 %) patients who did develop disease progression, median duration between initial diagnosis and progression was 8.7 months (range 1.5–148.2).

### EBV-Related Prognostic Factors

All EBV markers were included in survival analysis (see Table 3). EBER-positive and EBER-negative groups did not differ significantly in terms of OS (median OS 29.0 months vs not reached; *P* = 0.114). However, EBER-positive cases had significantly shorter TTP [18.0 months vs not reached (NR); *P* = 0.032]. Noteworthy, no association with OS or TTP could be shown for LMP1.

**Table 3:** Analysis of prognostic factors for survival and progression risk

Parameter	Overall survival		Time-to-progression	
	Median, mo	*P*	Median, mo	*P*
EBV-related factors				
EBER ISH				
Positive	29.0	0.114	**18.0**	**0.032**
Negative	NR		**NR**	
LMP1				
Positive	NR	>0.10	21.0	>0.10
Negative	NR		NR	
VCA IgG/IgM				
Positive	16.5	>0.10	NR	>0.10
Negative	90.5		6.5	
EA IgG				
Positive	NR	>0.10	NR	>0.10
Negative	90.5		NR	
EBNA IgG/IgM				
Positive	NR	>0.10	NR	>0.10
Negative	90.5		NR	
VCA/EA/EBNA IgG				
Positive	90.5	>0.10	NR	>0.10
Negative	16.5		7.1	
VCA/EA/EBNA IgM				
Positive	NR	>0.10	NR	>0.10
Negative	90.5		NR	
Other prognostic factors				
Age				
≥75	**35.0**	**0.008**	NR	>0.10
<75	**NR**		NR	
Ann Arbor stage				
1–2	**NR**	**0.007**	**NR**	**0.025**
3–4	**75.0**		**93.0**	
B symptoms				
Present	**75.5**	**0.002**	**93.0**	**0.007**
Absent	**NR**		**NR**	
Mediastinal bulk >50 mm				
Yes	**34.4**	**0.010**	11.1	>0.10
No	**NR**		NR	
BM involvement				
Present	**18.5**	**0.009**	NR	0.333
Absent	**NR**		NR	
ECOG PS				
0–1	**NR**	**0.002**	NR	0.857
2–4	**35.8**		NR	
LDH				
≥ULN	**NR**	**0.015**	NR	0.267
<ULN	**NR**		NR	
IPI				
0–2	**NR**	**0.001**	NR	0.929
3–4	**29.0**		NR	
Rituximab				
Yes	**NR**	**0.011**	**NR**	**0.021**
No	**34.8**		**65.0**	
IHC subgroup				
GCB	**NR**	**0.015**	**NR**	**0.034**
Non-GCB	**68.0**		**NR**	
Necrosis				
Yes	**12.0**	**0.010**	NR	0.883
No	**NR**		NR	
CR1				
Yes	**NR**	**0.000**	**NR**	**0.000**
No	**18.4**		**7.2**	

Associations with *P* ≤ 0.05 are written in bold

*EBV* Epstein–Barr virus, *EBER ISH* EBV-encoded small RNA in situ hybridization, *LMP1* latent membrane protein 1, *VCA* viral capsid antigen, *EA* early antigen, *EBNA* EBV nuclear antigen, *BM* bone marrow, *ECOG PS* Eastern Cooperative Oncology Group performance status scale, *LDH* lactate dehydrogenase, *ULN* upper limit of normal, *IPI* international prognostic index, *IHC* immunohistochemical, *GCB* germinal center B cell subtype, *CR1* complete response after first-line treatment, *NR* not reached, *mo* months

**Fig. 4:**
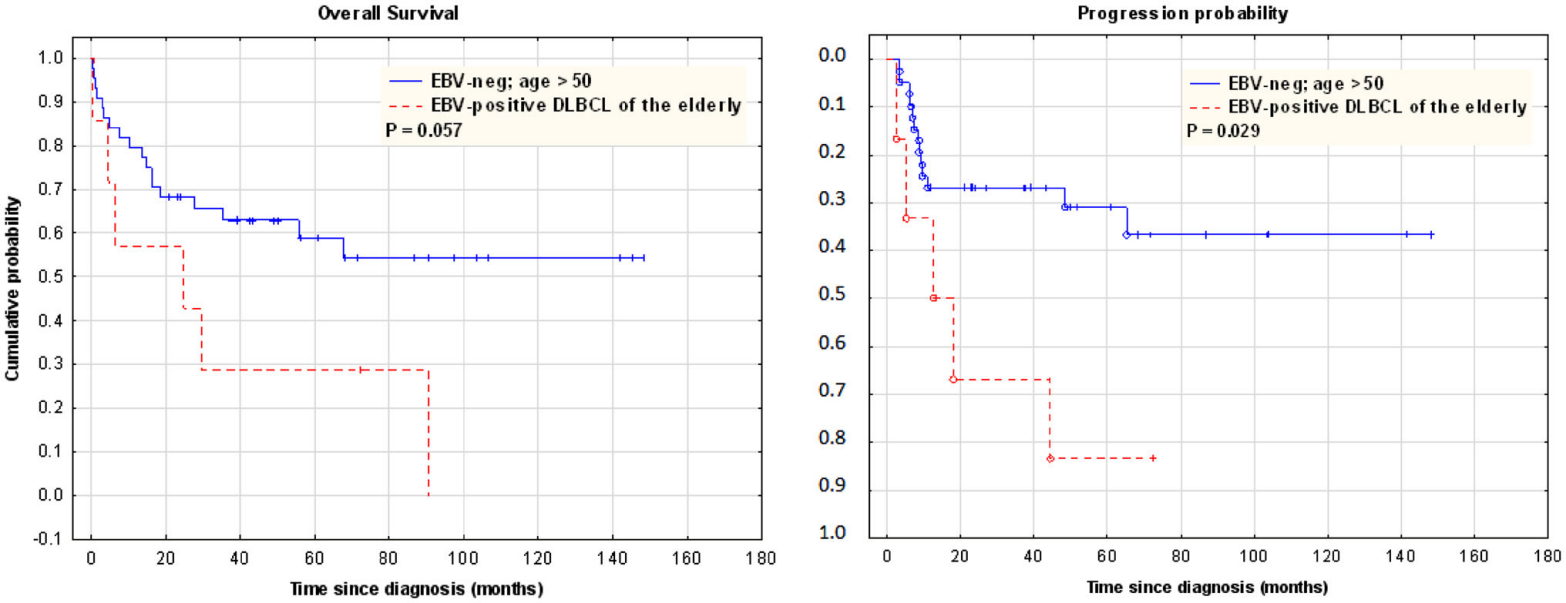
Overall survival and progression probability in EBV-positive DLBCL of the elderly vs aged-matched EBV-negative DLBCL

Subsequently, cases were compared with age-matched EBV-negative DLBCL population. The EBV-positive DLBCL of the elderly subgroup was characterized by a trend towards worse OS (median OS 24.5 months vs NR; *P* = 0.059), and a significantly higher progression probability (median TTP 12.5 months vs NR; *P* = 0.029) (see Fig. 4a, b).

No single EBV-specific antibody was associated with survival parameters. Furthermore, no impact on survival could be documented if antibodies were grouped according to class (IgG or IgM irrespective of antigen specificity) or antigen specificity (VCA/EA/EBNA-1 irrespective of type), as well as according to acute or chronic infection profiles (see above).

Subsequently, the study group was examined for other non-EBV-related prognostic factors (see Table 3). In OS analysis, factors significantly associated with longer survival were age under 75 years (*P* = 0.008), Ann Arbor stage ≤2 (*P* = 0.007), absence of B symptoms (*P* = 0.002), absence of bone marrow involvement (*P* = 0.009), performance status according to ECOG 0–1 (*P* = 0.002), serum baseline LDH activity below the upper limit of normal (*P* = 0.015), risk according to IPI ≤ 2 (*P* = 0.001), treatment with rituximab (*P* = 0.011), bulky mediastinal mass >50 mm in diameter (*P* = 0.010) as well as GCB immunohistochemical subtype (*P* = 0.015) and presence of necrosis in histologic tumor specimens (*P* = 0.010).

Complete response rate after first-line treatment in the study population was 48.0 %. Complete response rate was not associated with any of the EBV-related markers.

## Discussion

In the present study we described EBV infection and its prognostic impact in a sample of Polish DLBCL patients with special emphasis on the clinicopathologic characteristics of EBV-positive DLBCL of the elderly. The analysis included EBV serologic and histologic markers available both for pathologists and clinicians.

We set the threshold for EBER positivity at 5 % of malignant cells, and the observed range of positive cells was between 5 and 40 %. In the study of d’Amore et al. (1996) above 75 % of EBER-positive B cell non-Hodgkin lymphomas (B-NHL) contained only few scattered EBER-positive cells. Other authors set the limit for EBER positivity at 10–20 % (Gibson and Hsi 2009; Park et al. 2007; Ok et al. 2014). In fact, there are no uniform criteria for EBER positivity. The lower threshold may be more appropriate taking into consideration the morphological heterogeneity of EBV-positive DLBCL, which encompasses both monomorphic variants with dense cellular infiltrates and polymorphic variants with few scattered neoplastic cells (Ok et al. 2013). Thus, assuming higher thresholds for EBER positivity would cause inappropriate exclusion of polymorphic variants and omission of EBV-positive malignant subclones. On the other hand, low fraction of EBER-positive cells may be clonally unrelated or represent a late infection in lymphomagenesis (Hofscheier et al. 2011). Nevertheless, we were able to show the prognostic significance of minor EBV-positive fractions in DLBCL (see also below), strongly arguing in favor of the assumed 5 % threshold.

In comparison with values published worldwide, our results (12.2 % of EBER-positive DLBCL) are located above the median. EBER positivity was reported in 0 % (0/90) of DLBCL cases in first American series (Gibson and Hsi 2009), 4.0 % in large DLBCL cohort in USA (Ok et al. 2014), 5.3 % (18/340) of DLBCL cases in Turkey (Uner et al. 2011), 7 % (25/374) of non-Hodgkin lymphomas (NHL) in Danish registry (d’Amore et al. 1996), 9 % (34/380) of Korean DLBCL cases (Park et al. 2007), 12.7 % (9/71) of NHL patients in Pakistan (Ishtiaq et al. 2013) and 18 % (8/44) of DLBCL cases in Kuwait (Al-Humood et al. 2014). It should be noted that the frequency of EBV-positive DLBCL, as well as EBV-positive DLBCL of the elderly specifically is dependent on the chosen threshold for EBER positivity and thus makes geographical comparisons questionable (Wada et al. 2011). Thus, higher incidence of EBV-positive DLBCL in our study is directly correlated with the assumed EBER-positivity threshold and cannot be interpreted as an isolated observation.

In the present study, we were not able to detect any significant associations between histologic markers of EBV infection (EBER and LMP1) and a wide set of clinical and biological parameters (see Table 1). In the Korean study (*n* = 380), EBV-positive DLBCL cases were characterized by higher frequency of clinical risk factors (Park et al. 2007). However, in an American study, age and extranodal localization were not related with EBV infection in the overall DLBCL population, nor in the EBV-positive DLBCL of the elderly subpopulation (Hoeller et al. 2010). In another study on Kuwaiti population, EBV-positive DLBCL was not associated with clinical risk factors either (Al-Humood et al. 2014). Similarly, in the Danish registry of 374 B cell NHL (74 cases of DLBCL), no correlation was seen between EBER positivity and patients’ clinical characteristics (d’Amore et al. 1996).

The discordant results of LMP1 and EBER staining in our study should be interpreted with caution. The predominance of EBER-positive LMP1-negative cases may be explained by EBV latency type I. However, we did not perform EBNA IHC staining and thus we were not able to unambiguously detect the EBV latency type. The Danish group found LMP1 expression in only 20 % of EBER-positive B-NHL cases (d’Amore et al. 1996), though in recent Asian and American studies on EBV-positive DLBCL of the elderly, latency type III was characterized as most frequent (Gibson and Hsi 2009; Oyama et al. 2007). To confirm our results, more detailed genetic analyses of EBV gene expression pattern are required. We found a small subset of cases (*n* = 3) with concomitant positive LMP staining and absence of EBER in ISH analysis. However, taking into consideration that EBER is constantly positive in all described EBV latency phases, these cases cannot be regarded as EBV-infected and should be interpreted as unspecific staining.

The study included an analysis of EBV-specific antibodies, which disclosed lack of associations between serologic and pathologic EBV markers. Moreover, EBV-specific antibodies, irrespective of class and specificity, had no prognostic value. An interesting observation is the disappearance of EBNA IgG in older DLBCL patients with preservation of VCA IgG. Secondary EBNA IgG negativity in chronic EBV infection is described in immunocompromised states like HIV infection, solid organ transplantation and malignancies (Riddler et al. 1994; Vetter et al. 1994). It was also described by Oyama et al. (2007) in the initial study on EBV-positive DLBCL of the elderly. Thus, loss of EBNA IgG may be a sign of reduced capacity to control the virus in elderly DLBCL patients.

Our data show a very high EBV seropositivity rate in the population of Polish patients with DLBCL, reaching 97.2 % (frequency of VCA IgG). This result is comparable with other populations, for example, British pregnant women with seroprevalence of 94 % (Pembrey et al. 2013) or patients with inflammatory bowel diseases who achieve seroprevalence of 100 % by the age of 40 (Linton et al. 2013).

In the subset of EBV-positive DLBCL of the elderly, the frequency of extranodal localization was 57 %, lying between 69 % described in Asian populations (Oyama et al. 2007) and 0–25 % in Western studies (Gibson and Hsi 2009; Hoeller et al. 2010). The occurrence of both monomorphic and polymorphic morphology as well as occasional presence of Reed–Sternberg-like cells and plasma cell infiltrates seen in our series was also described by others (Gibson and Hsi 2009; Hoeller et al. 2010; Ok et al. 2013; Uner et al. 2011). EBV-positive DLBCL of the elderly cases had higher CD30 and lower BCL-6 expression, which was also noted by others. Hoeller et al. (2010) reported 50 % CD30-positive tumor cells in EBV-infected DLBCL in comparison with 4 % CD30-positive cells in EBV-negative cases and Oyama et al. (2007) showed 75 and 13 % of CD30-positive cells in EBV-positive and negative cases, respectively (*P* < 10^−5^). Al-Humood et al. (2014) reported lower BCL-6 expression in association with EBV (25 vs 55.5 % in EBV-positive vs negative cases, respectively; *P* = 0.01).

Among serological and pathological EBV markers, only EBER status was associated with prognosis. When comparing cases with the diagnosis of EBV-positive DLBCL of the elderly with age-matched EBV-negative DLBCL patients, the negative impact on progression risk and overall survival was accentuated. This observation suggests that EBER positivity confers poor prognosis irrespective of age. These data are consistent with the results of earlier studies. In the Korean study, EBER positivity was associated with both inferior OS (35.8 months vs median OS not reached, EBER positive vs EBER negative; *P* = 0.026) and PFS (12.8 vs 35.8 months, EBER-positive vs EBER-negative; *P* = 0.018) (Park et al. 2007). Japanese investigators found median OS of 24 months vs not reached in EBV-positive DLBCL of the elderly vs EBV-negative DLBCL, respectively; *P* < 10^−5^ (Oyama et al. 2007). The poor prognostic impact of EBV may result from specific activation of NF-κB pathway through overexpression of transcription factor STAT3 (Ok et al. 2014).

However, Danish Lymphoma Study Group reported that EBER status did not have any prognostic influence in combined analysis of B cell NHL (7-year survival of EBER-negative vs EBER-positive cases, 41 vs 36 %; median survival, 4.4 vs 2.9 years; *P* = 0.74), as well as in sub-analysis of intermediate to high-grade B-NHL, without specific analysis in DLBCL (7-year survival of EBER-negative vs EBER-positive cases, 37 vs 38 %; median survival, 2.8 vs 2.7 years; *P* = 0.85) (d’Amore et al. 1996). Moreover, a study by Ok et al. (2014) found no correlation between EBER positivity and prognosis in DLBCL and pointed to poor survival of CD30^+^ EBER^+^ cases. All patients in this study received treatment with rituximab. The low rate of rituximab treatment in our EBER-positive cases may explain the divergent survival results. In the present study, we were not able to detect any survival differences according to CD30 status both in the entire population and EBV-positive cases.

In the entire population of our study we were able to confirm the prognostic impact of generally recognized clinical risk factors, indicating comparability of our results with the published literature (Shipp et al. 1993). We did observe the prognostic significance of immunohistochemical cell of origin classification according to Hans et al. (2004). Other histological poor-risk feature was the presence of necrosis in biopsy material. However, in contrast to other reports, we did not observe a prognostic impact of CD5, CD30 and BCL-2 expression.

The main obstacle to interpret our survival data is the low number of EBV-positive cases, as well as slightly uneven distribution of rituximab therapy in EBV-positive and EBV-negative cases (this heterogeneity did not reach statistical significance: 33 vs 71 %, respectively; *P* > 0.10). Though, it cannot be ruled out that survival differences observed in our patients are biased by this factor. Rituximab-treated EBER-positive patients in our study appeared to fare better than EBER-positive patients not receiving rituximab. This notion is in-line with the results of retrospective Japanese study assessing rituximab-based chemotherapy, which showed similar survival irrespective of EBV status (Ahn et al. 2013), and with the results of American study by Ok et al. (2014).

In conclusion, the study introduces new data on the epidemiology of EBV-positive DLBCL of the elderly in the central-European population, confirming the prognostic significance of EBER status in Caucasians, which is especially marked in IPI low-risk and rituximab-naïve subgroups. The study shows the absence of associations between serologic and histological EBV markers.
